# Anti-Inflammatory Dipeptide, a Metabolite from Ambioba Secretion, Protects Cerebral Ischemia Injury by Blocking Apoptosis Via p-JNK/Bax Pathway

**DOI:** 10.3389/fphar.2021.689007

**Published:** 2021-06-18

**Authors:** Qian Zhang, Jinwei Dai, Zhibing Song, Yuchen Guo, Shanshan Deng, Yongsheng Yu, Tiejun Li, Yuefan Zhang

**Affiliations:** ^1^ School of Medicine, Shanghai University, Shanghai, China; ^2^ College of Pharmacology, Anhui University of Chinese Medicine, Hefei, China; ^3^ Department of Pharmacology, School of Life Science and Biopharmaceutics, Shenyang Pharmaceutical University, Shenyang, China

**Keywords:** cerebral ischemia, apoptosis, anti-inflammation dipeptide, neuroprotection, oxygen-glucose deprivation/reperfusion

## Abstract

MQ (l-methionyl-l-glutamic acid), anti-inflammatory dipeptide, is one of the metabolites of monocyte locomotion inhibitory factor, a thermostable pentapeptide secreted by *Entamoeba histolytica*. Monocyte locomotion inhibitory factor injection has been approved as an investigational drug for the potential neural protection in acute ischemic stroke. This study further investigated the neuroprotective effect of MQ in ischemic brain damage. Ischemia-reperfusion injury of the brain was induced in the rat model by middle cerebral artery occlusion. 2,3,5-triphenyltetrazolium chloride staining assay was used to measure cerebral infarction areas in rats. Laser Doppler measurement instrument was used to detect blood flow changes in the rat model. Nissl staining and NeuN staining were utilized to observe the numbers and structures of neuron cells, and the pathological changes in the brain tissues were examined by hematoxylin–eosin staining. Terminal deoxynucleotidyl transferase deoxyuridine triphosphate nick end labeling (TUNEL) staining was used to assess cell apoptosis. The changes in oxidative stress indexes, superoxide dismutase and malondialdehyde (MDA), were measured in serum. Methyl thiazolyl tetrazolium was used to measure the survival rates of PC12 cells. Flow cytometry assessed the apoptosis rates and the levels of reactive oxygen species. Real-time PCR was used to evaluate the mRNA expression levels, and Western blotting was used to analyze the changes in protein levels of p-JNK, Bax, cleaved Caspase3. We revealed that MQ improved neurobehavior, decreased cerebral infarction areas, altered blood flow volume, and the morphology of the cortex and hippocampus. On the other hand, it decreased the apoptosis of cortical neurons and the levels of MDA, and increased the levels of superoxide dismutase. *In vitro* studies demonstrated that MQ enhanced the cell survival rates and decreased the levels of reactive oxygen species. Compared to the oxygen-glucose deprivation/reperfusion group, the protein and mRNA expressions of p-JNK, Bax, cleaved Caspase3 was decreased significantly. These findings suggested that MQ exerts a neuroprotective effect in cerebral ischemia by blocking apoptosis via the p-JNK/Bax pathway.

## Introduction

Ischemic stroke, with high morbidity, high disability, and high mortality, accounts for about 80% of all stroke cases and causing severe nerve injuries (Poustchi et al., 2021). Prolonged cerebral ischemia leads to irreversible neuronal death, and reperfusion after ischemia results in brain injury, as assessed in animal studies or clinical trials (Lapchak and Zhang, 2017; Wu et al., 2020). Apoptosis is a regulated and programmed cell death that plays a crucial role in cerebral ischemia-induced brain injury in human and animal models (Sairanen et al., 2009; Liang et al., 2014). Hitherto, there are only a few effective drugs to treat ischemia/reperfusion (I/R) injury, especially for neural damage (Liang et al., 2011). Therefore, finding the anti-neuroapoptosis drugs for the treatment of ischemic stroke is clinically significant.

Animal medicine is a vital part of traditional Chinese medicine, such as *earthworm* and *Leech*. Peptides from animal medicine have various pharmacological activities. *Entamoeba histolytica*, an anaerobic pathogenic enteric protozoan and tissue-invasive parasite (Lee et al., 2019), is responsible for amoebic dysentery and amoebic liver abscess, resulting in human suffering and death (Soares et al., 2019). The pathogen is commonly found in low-resource regions, including Africa, India, Mexico, and parts of central/south America, and Southeast Asia (Wingfield et al., 2018). MLIF, a natural product derived from *Entamoeba histolytica* axenic cultures (Kretschmer et al., 1989; Rico et al., 1998), has been approved as an investigational drug for the potential neural protection in acute ischemic stroke (Liu et al., 2018). It was first identified as an anti-inflammatory peptide that inhibited the migration of monocytes (Kretschmer et al., 2001; Barrientos-Salcedo et al., 2009; Silva-Garcia and Rico-Rosillo, 2011) and the expression of interleukin (IL)-1β, IL-5, IL-6, and interferon (IFN)-γ and increased the expression of IL-10 in macrophages (Utrera-Barillas et al., 2003; Rojas-Dotor et al., 2006; Silva-Garcia et al., 2008). Subsequent studies showed that MLIF participated in other biological effects, such as expression of immunomodulatory genes (Silva-Garcia et al., 2008; Rojas-Dotor et al., 2018), cell proliferation, extracellular matrix formation, angiogenesis, and axon guidance. In a previous study, we discovered that MLIF exerts a protective effect on the vascular system by acting on eukaryotic elongation factor1A1 (eEF1A1) to upregulate the expression of vascular endothelial nitric oxide synthase (eNOS) and reduce the level of ICAM-1 and VCAM-1 (Zhang et al., 2012). In addition, MLIF decreased cerebral infarction areas and the level of malondialdehyde (MDA), myeloperoxidase (MPO), tumor necrosis factor-alpha (TNF-α), and IL-1β and increased the level of superoxide dismutase (SOD) (Yao et al., 2011). It also acted on eEF1A2 and the p-JNK/p53 pathways to protect the nerves (Zhu et al., 2016). Both native and synthetic MLIF presented selective anti-inflammatory properties, thereby inhibiting the human monocyte locomotion and respiratory burst and altering the expression of both anti-inflammatory and pro-inflammatory cytokines (Liu et al., 2018). However, due to the disadvantages of high synthesis cost, the related structure of MLIF is under continuous improvement. Finally, another peptide, an anti-inflammatory dipeptide, was found.

MQ is one of the metabolites of MLIF, which consists of methionine and glutamine. Endogenous peptides play a critical role in human function. A previous study (Baek et al., 2014) stated that carnosine, an endogenous dipeptide, converted LC3-I to LC3-II and reduced the expression of phosphorylated mTOR/p70S6K in the ischemic brain by improving brain mitochondrial function and mitophagy signaling. The dimeric dipeptide mimetic GK-2 prevented H_2_O_2_^−^ or glutamic acid-induced destruction of hippocampal neurons and improved cognitive and memory functions to exert neuroprotective effects (Povarnina et al., 2013). Additionally, it prevented delayed neuronal damage and axonal degeneration and reduced TBI-related disruptions of brain functions (Genrikhs et al., 2018).

Therefore, the present study investigated the neuroprotective effect of MQ by blocking apoptosis via p-JNK/Bax pathway *in vitro* and *in vivo*.

## Materials and Methods

The experimental procedures were conducted according to the ARRIVE guidelines. Adult male Sprague–Dawley (SD) rats (220–270 g), purchased from the Changzhou Cavens Experimental Animals Co., Ltd (Changzhou, China), were housed in controlled conditions and given rat chow and tap water ad libitum. The surgery was executed after one week. The animal experiments were conducted under the supervision of the Animal Ethics Committee of Shanghai University (Shanghai, China), according to the National Institutes of Health Guide for the Care and Use of Laboratory Animals. We also made efforts to meet the principle of “3Rs” (Reduction, Refinement, and Replacement), according to the law.

MQ was synthesized by the Chinese Peptide Company (Hangzhou, China), with purity >98% determined by HPLC. MLIF were synthesized by the Chinese Peptide Company (Hangzhou, China), with purity >98%. 2,3,5-triphenyl-tetrazolium chloride (TTC) was purchased from Sigma (St. Louis, MO, United States). SOD and MDA kits were bought from Nanjing Jiancheng Bioengineering Institute (Nanjing, China). Reactive Oxygen Species (ROS) kit, Annexin V- fluorescein isothiocyante kit, TUNEL kit, and 2-(4-Amidinophenyl)-6-indolecarbamidine dihydrochloride (DAPI) were obtained from Beyotime (Shanghai, China). p-JNK, Caspase3, Bax, GAPDH, NeuN antibodies were purchased from Cell Signaling Technology (CST, Danvers, MA, United States). JNK inhibitor (SP600125) was purchased from Selleck (Shanghai, China). BCA protein kit was bought from Thermo Scientific (Waltham, MA, United States).

### Middle Cerebral Artery Occlusion (MCAO)

Cerebral I/R was induced by MCAO, as described previously (Fan et al., 2011; Zhang et al., 2012). The rats were anesthetized with an intraperitoneal injection of 10% chloral hydrate (3 mL/kg). Then, the neck skin incision was scissored, and the tissues were bluntly dissected with tweezers to expose the left common carotid artery (CCA). The internal carotid artery and the external carotid artery were separated, and the external carotid artery was ligatured. After a small incision was made in the proximal CCA, the occlusion line (0.26 mm) was inserted (18 mm) into the internal carotid artery through the CCA, thereby occluding the origin of the MCA. The body temperature was maintained at 37°C with warm light for 2 h, following which the occlusion line was pulled out with the curved tweezer for reperfusion. After 24 h, the rats were sacrificed for subsequent experiments.

### Experimental Groups and Drug Treatment

The experimental rats were randomly divided into the following groups: sham operation (sham + saline solution), cerebral I/R (model + saline solution), I/R + MQ (3 mg/kg) hydrochloric, and I/R + MQ (1 mg/kg) hydrochloric. At 30 min post-MCAO surgery, MQ and normal saline were administered through the caudal vein.

In the comparison experiment of MQ and MLIF, the experimental rats were randomly divided into sham group + saline solution, model group + saline solution, I/R + MQ (3 mg/kg) hydrochloric group, and I/R + MLIF (3 mg/kg) hydrochloric group. At 30 min post-MCAO surgery, MQ, MLIF, and normal saline were administered through the caudal vein.

### Cerebral Blood Flow (CBF) Monitoring

The rat was fixed on the stereotaxic instrument after anesthesia, and a scalp wound was made along the centerline. Then, a 30% hydrogen peroxide solution was applied to the scalp to remove the periosteum. The dental drill was used to drill the skull until the meninges were visible behind the anterior fontanelle at 3 mm or near the midline at 3.5 mm. The laser sensor was mounted on the drill hole with glue. Subsequently, Doppler blood flow meter monitoring was used to evaluate CBF at the following time points: (a) 30 min before cerebral ischemia, (b) 30 min after cerebral ischemia, and (c) 30 min after cerebral I/R. CBF decrease (%) = (CBF_c_ − CBF_b_)/CBF_a_ × 100%.

### Neurological Deficit

The neurological function was assessed, as described previously (Longa et al., 1989; Fan et al., 2011) on a five-point scale system: 0, no neurological deficit; 1, failure to extend left forepaw fully; 2, circling to the contralateral side; 3, leaning to the contralateral side; 4, no initiative in walking with consciousness (Gu et al., 2016). The high scores indicated severe neurological functional damage. The investigators were blinded to both the type of surgery and the treatment.

### Quantitative Analyses

Image J software (National Institute of Health) was used for quantitative analyses of the data. The quantitative analyses were conducted by two independent examiners blinded to the treatment, and the average of the data was calculated.

### Measurement of Infarct Volume

The rats were anesthetized with intraperitoneal administration of 10% chloral hydrate (3 mL/kg). The brain tissues were excised and immediately placed at −20°C for 15 min. Then, the brain tissue was cut evenly into six coronary sections and placed in the 1% TTC for 15 min at 37°C and fixed in 4% paraformaldehyde. The researcher measuring the infarct area was blinded to the treatment group. The infarct volume was quantified and analyzed using Image J software (Li et al., 2020).

### H&E Staining

Rats were deeply anesthetized, and the heart was perfused with saline. The brain was removed after perfusion, followed by 4% paraformaldehyde fixation for 24 h. Subsequently, the brain tissues were sectioned at 5-μm thickness using a paraffin slicing machine. Then, the sections were dewaxed with xylene and dehydrated with graded alcohol. Finally, the sections were stained with H&E, as described previously (Feng et al., 2020), and observed under an optical microscope (×400). The sections used in the following staining were observed by two experienced pathologists blinded to the groups.

### Nissl Staining

Brain tissue was embedded in paraffin and sliced into 10-μm-thick coronal sections. Next, the slices were dewaxed, rehydrated (Shen et al., 2021), stained for 10 min in methyl violet solution, 2% acetic acid for 6 s, followed by anhydrous ethanol for 30 s and observed under a fluorescent microscope; the neuronal cells were counted and analyzed using Image J.

### Terminal Deoxynucleotidyl Transferase (TdT) Deoxyuridine Triphosphate Nick-End Labeling (TUNEL) Staining

The apoptotic brain cells were counted using the TdT-mediated dUTP-streptavidin-HRP method. Then, the sections were dewaxed as described above, and the nucleases were inactivated with the proteinase K. The endogenous peroxidase activity was quenched by adding 3% Triton X-100. The sections were blocked with 1% bovine serum albumin (BSA) and permeabilized with 3% Triton X-100 for 1 h, followed by incubation with the TUNEL detection liquid. Subsequently, the sections were incubated with streptavidin-horseradish peroxidase (HRP) and observed under a microscope at ×400. The number of apoptotic cells in three different fields of non-overlapping brain tissue was counted for further analysis.

### NeuN Staining

The sections were dewaxed with xylene and dehydrated with graded alcohol. The antigen was retrieved using proteinase K for 30 min. Then, the sections were incubated with primary antibody (NeuN, 1:20) at 4°C overnight, mounted in glycerin and phosphate-buffered saline (PBS) (1:1), and observed under laser confocal microscopy (×400).

### SOD and MDA Assay

The blood serum was collected by centrifugation at 12,000×*g* for 10 min at 4°C. The BCA Protein Assay Kit (Tiangen Biotech, Beijing, China) was used to determine the protein concentration of serum. The MDA and SOD levels were detected using the MDA kit (TBA colorimetry) and the SOD kit (WST-1 method). The OD values of the serum were measured on a microplate reader (Bio-Rad, CA, United States) at 532 and 450 nm.

### Cell Culture

Rat adrenal pheochromocytoma PC12 cells were obtained from the Cell Bank of Chinese Academy of Sciences (Shanghai, China) and cultured in Dulbecco’s modified Eagle medium (DMEM) (Hyclone, Logan, UT, United States) with 100 μg/mL penicillin-streptomycin solution (Thermo Scientific, Waltham, MA, United States) and 10% fetal bovine serum (FBS; Gibco, Carlsbad, CA, United States) at 37°C under 5% CO_2_ and 95% humidified atmosphere.

### Oxygen-Glucose Deprivation/Reperfusion (OGD/R) Model and MQ Treatment

Before OGD, the PC12 cells were washed three times with PBS and pretreated for 1 h with MQ (10 μg/mL) and diluted in PBS (0.001 μg/mL) with neurobasal and glucose-free DMEM. Then, the cells were placed in an oxygen-deficiency incubation device with 95% N_2_ and 5% CO_2_ for 2, 4, and 6 h at 37°C. For reperfusion, PC12 cells were cultured in a normal medium at 37°C for 12 h for subsequent experiments. A normal normoxia medium served as the control.

### Methyl Thiazolyl Tetrazolium (MTT) Assay

Cells were inoculated in 96-well culture plates at a density of 1 × 10^5^ cells/mL, and OGD/R was carried out as described above. Then, 20 μL MTT (5 mg/kg) was added to each well for 4 h at 37°C, and the reaction was quenched with 150 μL dimethyl sulfoxide (DMSO). The absorbance was measured at 490 nm on a microplate reader. The percentage of cell viability was calculated as follows: Cell viability (%) = absorbance value of sample/absorbance value of control × 100%.

### Annexin V-Fluorescein Isothiocyanate (FITC) Assay

To detect cell apoptosis, Annexin V-FITC assay was conducted according to the manufacturer’s protocol. Briefly, PC12 cells were collected by centrifugation at 1,000 *g* for 5 min at 4°C and stained with 200 μL Annexin V-FITC for 15 min and 10 μL propidium iodide (PI) at room temperature in the dark for 15 min. Then, the apoptosis rate of each group was detected by flow cytometry.

### ROS Assay

Flow cytometry was used to analyze the generation of intracellular ROS using a fluorescence probe 2′7′-dichlorodihydrofluorescein diacetate (DCFH-DA, Beyotime, China), which was diluted with serum-free medium (1:1,000). Then, the cells were added and the reaction incubated for 30 min at 37°C, followed by PBS washes before analysis (Xiang et al., 2019).

### Real-Time Quantitative Polymerase Chain Reaction (RT-qPCR)

The cells were lysed with RNAiso Reagent (TaKaRa, Shiga, Japan) to obtain total RNA. Reverse transcription reaction was carried out using an All-in-One cDNA Synthesis SuperMix assay kit (TaKaRa). The cDNA was synthesized in a 10 μL reaction containing 500 ng of total RNA, 2 μL of 5× qRT Super Mix, and RNase-free water at 42°C for 15 min or 85°C for 2 min. The PCR reaction (20 μL) consisted of 10 μL of 2× SYBR Green qPCR Master Mix kit (Bimake, Shanghai, China), 1 μL of cDNA, 1 μL of each primer, and 7 μL RNase-free water. The reaction conditions were as follows: initial denaturation at 95°C for 5 min, followed by 40 cycles of denaturation at 95°C for 5 s, primer annealing at 55°C for 30 s, and extension at 72°C for 30 s on a 7500 Real-Time PCR System (Applied Biosystems, Shanghai, China). The mRNA expression levels were evaluated using the 2^−ΔΔCq^ method. The primer sequences were as follows: *JNK* (Forward: 5′-GGG​CCT​TTT​TGC​TAC​AGG​GT-3′, Reverse: 5′-TTC​TTG​GTG​GAT​GCG​TCC​TG-3′), *Bax* (Forward: 5′-TGA​CGC​CTT​ATG​TGG​TGA​CT-3′, Reverse: 5′-TGA​TGT​ATG​GGT​GGT​GGA​GA-3′), *GAPDH* (Forward: 5′-ACC​ACA​GTC​CAT​GCC​ATC​AC-3′, Reverse: 5′-TCC​ACC​ACC​CTG​TTG​CTG​TA-3′), *Caspase3* (Forward: 5′-GAG​CTT​GGA​ACG​CGA​AGA​AA-3′, Reverse: 5′-TAA​CCG​GGT​GCG​GTA​GAG​TA-3′).

### Western Blot

Western blot was performed as described previously (Li et al., 2020; Si et al., 2020). The whole-cell lysate was prepared using M-PER Protein Extraction Reagent (Pierce, Rockford, IL, United States) supplemented with protease inhibitor cocktail. The protein concentration was measured using the BCA protein kit (Thermo). The protein lysate was resolved on 10% sodium dodecyl sulfate-polyacrylamide gel electrophoresis (10% SDS-PAGE), according to the molecular weight, and transferred to a nitrocellulose membrane. The membranes were blocked with 5% BSA in Tris-buffered saline with Tween-20 (TBST) at room temperature for 2 h before probing with the primary antibodies against p-JNK, Bax, cleaved Caspase-3, and GAPDH (1:1,000, CST) overnight at 4°C. The immunoreactive proteins were detected using enhanced chemiluminescence (ECL) (Thermo Scientific). All bands were quantified using Image J software.

### Statistical Analysis

All statistical analyses of experimental data were carried out using SPSS 17.0 (SPSS, Chicago, IL, United States). Data were reported as mean ± standard error of the mean (SEM). The differences among more than two groups were assessed using one-way analysis of variance (ANOVA) after the assessment of the normal distribution and homogeneity of variance of data, followed by Dunnett’s multiple comparison tests. *p* < 0.05 indicated statistically significant difference. Data were analyzed using Image J and Prism 8.0 (GraphPad Software, San Diego, CA, United States).

## Results

### MQ Increased Reperfusion CBF, Decreased Infarct Volume, and Neurological Deficits in MCAO Rats

**FIGURE 1:**
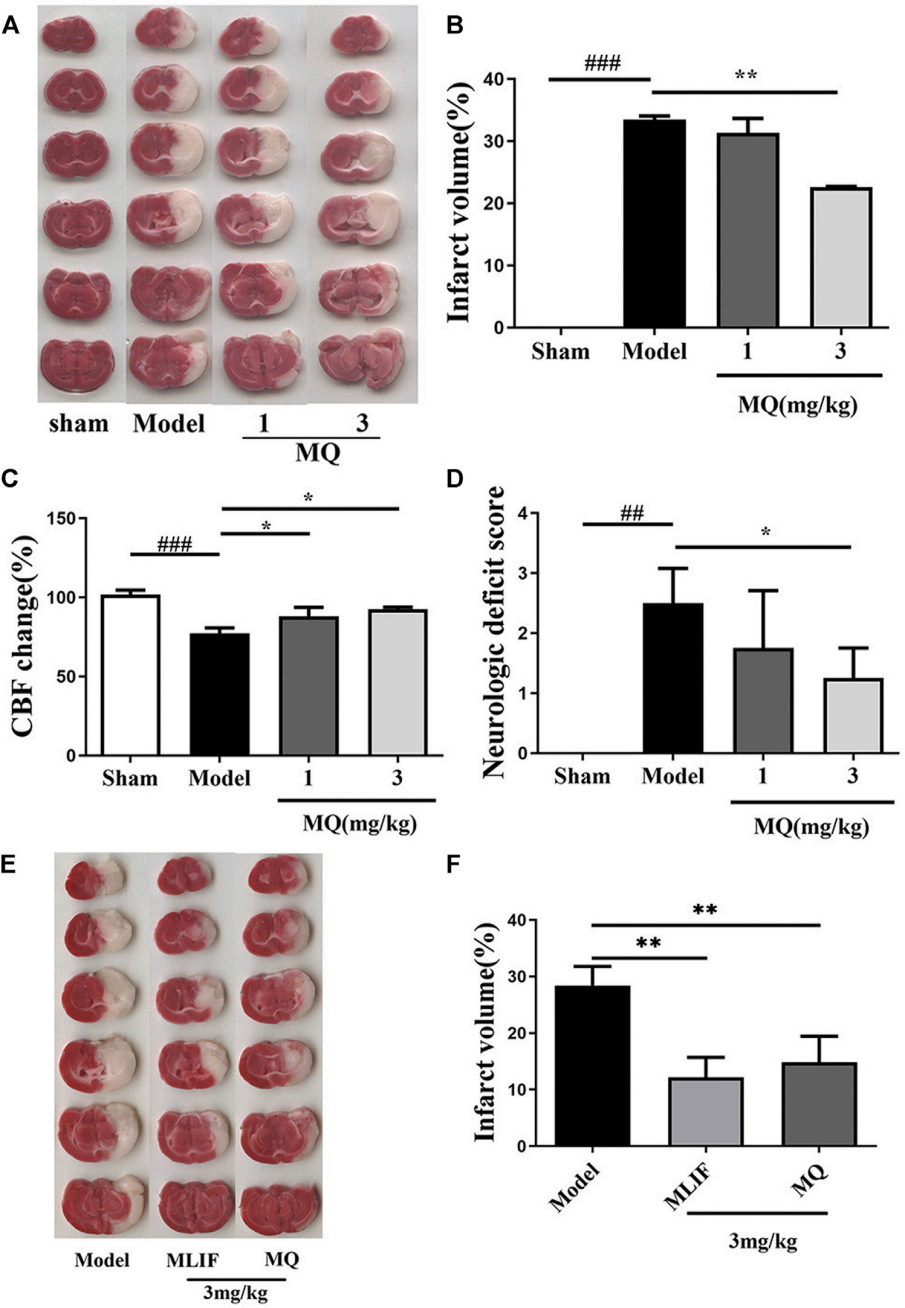
MQ increased reperfusion CBF, decreased infarct volume, and neurological deficits in MCAO rats; MLIF decreased infarct volume similar to MQ. **(A)** TTC staining was used to assess the infarct areas of the brain, **(B)** brain infarct areas were analyzed by Image J to estimate the infarct areas in the whole hemisphere after MCAO, **(C)** effect of MQ on the values of cerebral blood flow after cerebral I/R in rats, **(D)** effects of MQ on the neurological function after cerebral I/R in rats, **(E)** the infarct areas of the model group (28.42 ± 3.08%) increased dramatically compared to those of the sham group, and **(F)** the infarct areas of the MLIF and MQ (11.55 ± 3.81%, 14.85 ± 4.20%) decreased significantly compared to the model group. ^###^ *p* < 0.001, Model group vs. Sham group; *p < 0.05, **p < 0.01, MQ group vs. Model group, *n* ≥ 3.

To investigate the *in vivo* effect of MQ, we explored the ischemic brain model in SD rats. The cerebral infarction was estimated by TTC staining on the day following artery occlusion. As shown in Figure 1A, the infarct areas of the model group (33.46 ± 0.83%) increased significantly compared to those of the sham group (*p* < 0.001). The infarct area of the high-dose group (3 mg/kg) (22.60 ± 0.163%) decreased significantly (*p* < 0.01) compared to the model group (Figure 1B). The rate of cerebral blood flow in the rats was measured by a laser Doppler flow analyzer, and a significant increase was detected in the low-dose and high-dose groups compared to the model group (*p* < 0.05) (Figure 1C). The neurological functions in the rats were observed 24 h after cerebral I/R. The sham group did not show any neurological deficits, while the high-dose group showed a decreasing trend of neurological deficits compared to the model and low-dose groups (*p* < 0.05) (Figure 1D).

To compare the neuroprotective effect of MLIF to that of MQ, we assessed the ischemic brain model in SD rats. Cerebral infraction was estimated by TTC staining after the day of artery occlusion. As shown in Figure 1E, the infarct areas of the model group (28.42 ± 3.08%) increased dramatically compared to those of the sham group (*p* < 0. 01). The infarct area of the MLIF and MQ (3 mg/kg) (11.55 ± 3.81% and 14.85 ± 4.20%, respectively) decreased significantly (*p* < 0.01) compared to that of the model group (Figure 1F).

### MQ Reduced Cell Apoptosis of the Brain in MCAO Rats

**FIGURE 2:**
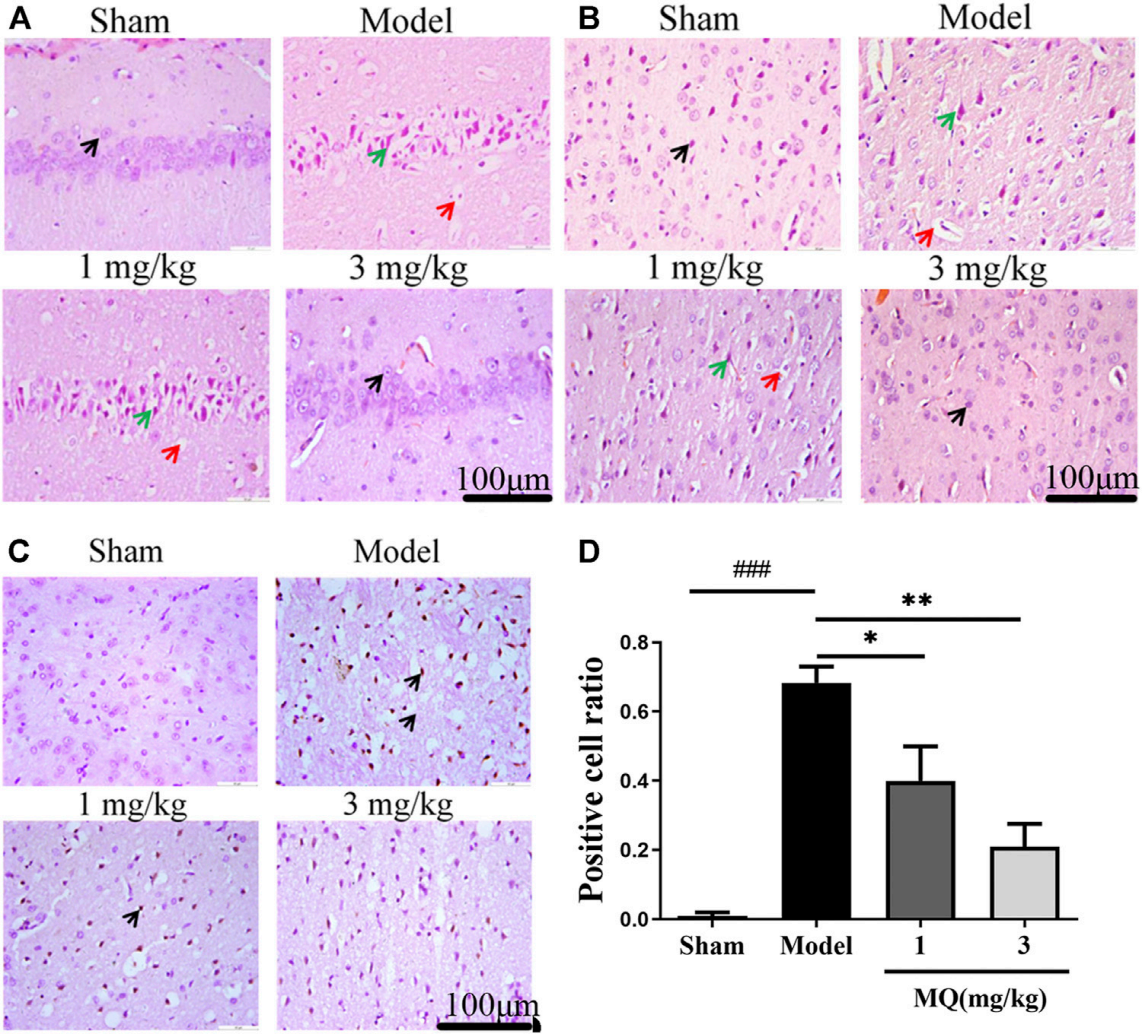
MQ reduced cell apoptosis of the brain in MCAO rats. **(A)** The hippocampi were stained with H&E (magnification ×400), **(B)** the cortices were stained with H&E (magnification ×400). Normal neurons (black arrow), atrophic neurons (green arrow), and cavitation edema neurons (red arrow), **(C)** the cortices were stained with TUNEL (magnification ×400) (D) The percentage of positive cells after I/R in different groups. ^###^ *p* < 0.001, Sham group vs. Model group; **p < 0.01, Model group vs. 3 mg/kg group; *p < 0.05, Model group vs. 1 mg/kg group, *n* = 3.

The morphological differences of SD rats were assessed by H&E staining one day after the operation (Figures 2A,B). We observed that the cell nucleus was full and clear, the structure was unbroken, and cells were arranged closely in the sham group, while the cell nucleus was shrunk and vacuoles appeared in the model and low-dose groups. Moreover, a large number of cells were detected in the cortex and the hippocampus in the high-dose group of MQ.

TUNEL staining was used to detect the apoptosis of the brain tissues, and the number of TUNEL-positive cells was calculated using Image J software. As shown in Figure 2D, no positive neurons were detected in the sham group. However, after treatment with MQ, the low-dose (*p* < 0.05) and the high-dose (*p* < 0.01) groups had fewer dark-brown positive neurons in the cortex compared to the model group. Additionally, we observed that MQ protects the nerve cells after cerebral ischemia injury by reducing apoptosis.

### MQ Alleviated Nerve Injury Induced by MCAO

**FIGURE 3:**
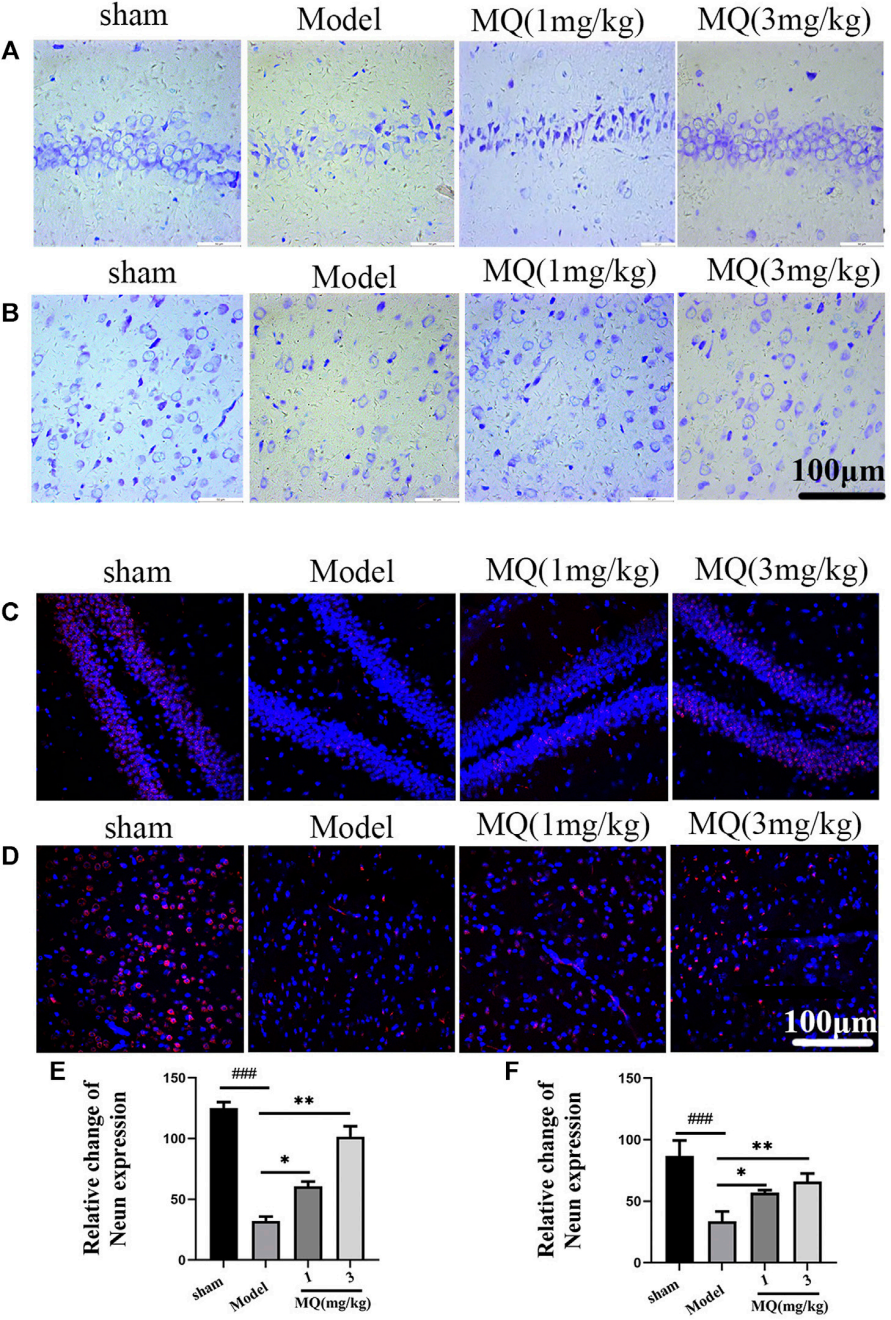
MQ alleviated the nerve injury induced by MCAO **(A)** The hippocampi were stained with Nissl (magnification ×400), **(B)** the cortices were stained with Nissl (magnification ×400), **(C)** the hippocampi were stained with NeuN (magnification ×400), **(D)** the cortices were stained with NeuN (magnification ×400), **(E)** number of nerve cells in hippocampi, **(F)** number of nerve cells in cortices. Dark brown-positive apoptotic cells (black arrow). ^###^ *p* < 0.001, ^##^ *p* < 0.01, Sham group vs. Model group; ***p < 0.001, **p < 0.01, *p < 0.05, MQ group vs. Model group, *n* = 3.

To further assess the neuroprotection of MQ, we performed Nissl staining to count neurons on the day after MCAO. The numbers and morphological changes in Nissl bodies were speculated as vital components reflecting the growth and development of the neurons and the material energy exchange and functions among nerve cells. As shown in Figure 3, the nerve cells were arranged orderly and did not show any damage in the sham group, while in the mode group, the cells were shrunken and disordered. Furthermore, interstitial spaces were detected in the cells, but the number decreased in the model group compared to the sham group (*p* < 0.001). After the treatment with high-dose dipeptide, the number and morphology of the nerve cells were improved significantly (*p* < 0.01).

NeuN staining revealed the modifications in nerve cells after cerebral ischemia. NeuN-positive cells were marked red, and DAPI-positive cells were marked blue. As shown in Figure 3, the number of NeuN-DAPI-positive cells (white arrow) in the high-dose group was more than the number in the model group (*p* < 0.01). These findings indicated that MQ alleviated nerve injury after ischemia.

### Effects of MQ on the Levels of SOD and MDA in MCAO Rats

**FIGURE 4:**
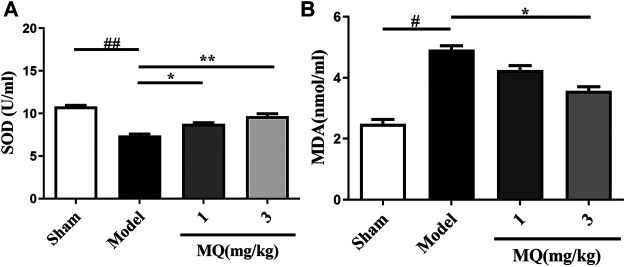
Effects of MQ on the levels of SOD and MDA in MCAO rats. **(A)** The activity of SOD after I/R in different groups, **(B)** the levels of MDA after I/R in different groups. **p < 0.01, Model group vs. 3 mg/kg group; **p < 0.01, Model group vs. 3 mg/kg group; *p < 0.05, Model group vs. 1 mg/kg group, *n* = 3.

Since oxidative stress plays a major role in MCAO injury, we investigated the effect of MQ on SOD activities and MDA levels in the serum. As shown in Figure 4A, an obvious decline was observed in the SOD levels in the serum of the model group compared to that of the sham group (*p* < 0.01). After treatment with MQ, the SOD levels increased subsequently; significant differences were observed in the low-dose (*p* < 0.05) and high-dose (*p* < 0.01) groups vs. the model group. On the other hand, a declining trend was noted in the MDA levels in the high-dose group compared to the model group (*p* < 0.05) (Figure 4B).

### MQ Decreased Cell Apoptosis in OGD/R-Induced PC12 Cells

**FIGURE 5:**
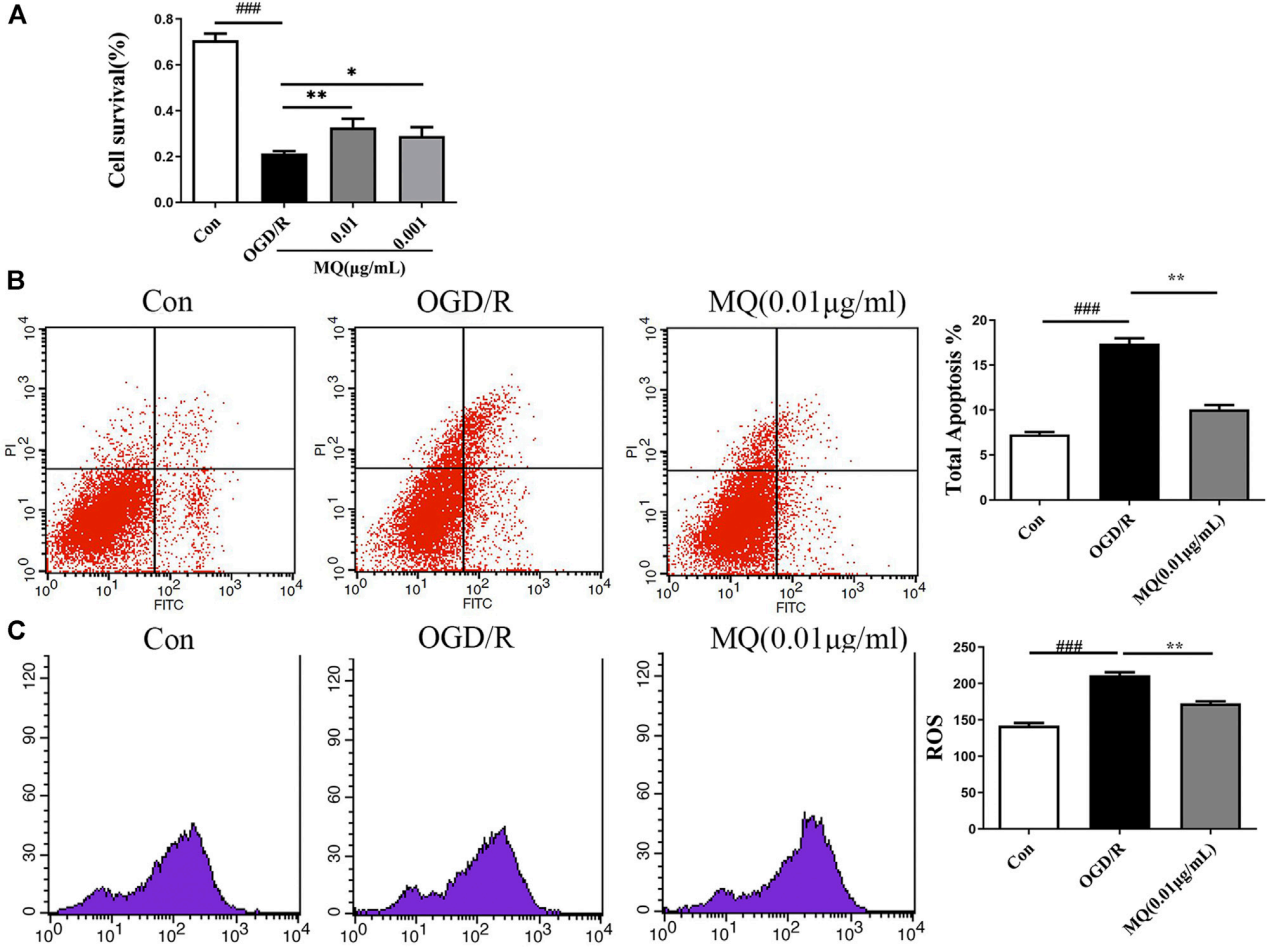
MQ decreased cell apoptosis in OGD/R-induced PC12 cells. **(A)** MTT was used to test PC12 cell viability, **(B)** flow cytometry data showed the percentage of total apoptotic cells compared to the OGD groups, **(C)** flow cytometry data showed the level of ROS in PC12 cells. MQ: 0.01 μg/mL; ^###^ *p* < 0.001, control group vs. OGD group, *p < 0.05, **p < 0.01, MQ group vs. OGD group, *n* ≥ 3.

To further evaluate the protective roles of MQ, we determined the effect of MQ on OGD/R-induced apoptosis in PC12 Cells. After OGD/R, MTT assay was used to detect the best time of OGD and the optimum concentration of MQ. We found that the cell survival rate was the lowest (32.81 ± 0.828%) after OGD treatment for 2 h. Figure 5A shows that the pretreatment with MQ (0.01 and 0.001 μg/mL) showed that the survival rate differed significantly compared to that of the OGD/R group (*p* < 0.01, *p* < 0.05). The results of the flow cytometry showed that the cells treated with 0.01 μg/mL MQ had a lower percentage of total apoptotic cells than the OGD group (*p* < 0.01; Figure 5B). Furthermore, the ROS level was decreased in PC12 cells treated with 0.01 μg/L MQ compared to that of the OGD/R group, as revealed by flow cytometry data (*p* < 0.01; Figure 5C).

### MQ Decreased Cell Apoptosis Via p-JNK/Bax Pathway

**FIGURE 6:**
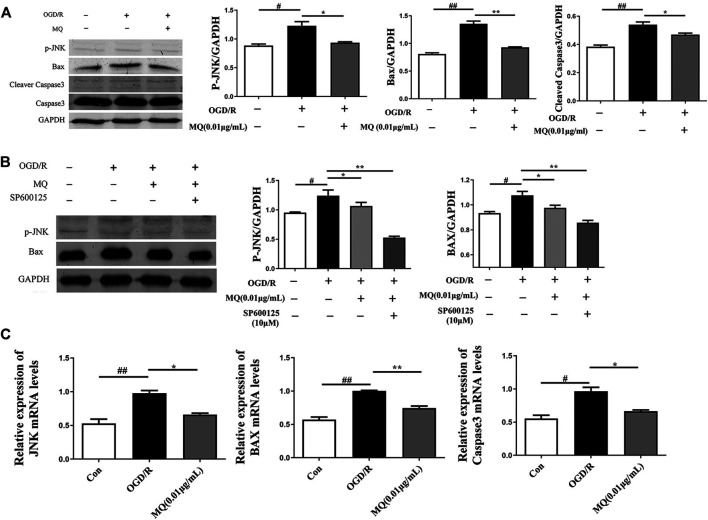
MQ decreased cell apoptosis via p-JNK/Bax pathway. **(A)** Protein expression of p-JNK, Bax, and cleaved Caspase3 levels in PC12 cells, as examined by Western blot, **(B)** representative protein expression of p-JNK and Bax treated with JNK inhibitor (SP600125), as examined by Western blot, **(C)** Effects of MQ on the mRNA levels of *JNK*, *Bax*, and *Caspase3* after OGD/R. ^**#**^ *p* < 0.05, ^**##**^ *p* < 0.01, OGD/R group vs. Con group; *p < 0.05, **p < 0.01, OGD/R group vs. MQ 0.01 μg/mL group; *n* = 3.

As shown in Figure 6A, the expression of p-JNK, Bax, and cleaved Caspase 3 proteins was significantly upregulated in the OGD/R group compared to the control group (*p* < 0.05, *p* < 0.01). However, the expression of p-JNK, Bax, and cleaved Caspase 3 declined after treatment with MQ compared to the OGD/R group (*p* < 0.05, *p* < 0.01, and *p* < 0.05, respectively). Compared to the normal control group, the level of p-JNK and Bax proteins was markedly higher (*p* < 0.05); whereas, the treatment with MQ and JNK inhibitors (SP600125) significantly decreased the level of these two proteins compared to the OGD/R group (*p* < 0.01).

We also observed that the mRNA levels of *JNK*, *Bax*, and *Caspase3* decreased after MQ treatment of PC12 cells. The results showed that MQ inhibited cell apoptosis through the p-JNK/Bax pathway.

## Discussion

Acute stroke therapy has significantly evolved over the last two decades. Intravenous chemical thrombolysis and intra-arterial mechanical thrombectomy have been utilized worldwide. However, the recovery of blood flow by thrombolytic therapy damaged brain tissues. On the other hand, inhibiting I/R injury is the most effective treatment strategy for alleviating the clinical symptoms (Jin et al., 2017; Koh and Park, 2017). Oxidative stress is involved in reperfusion (Lu et al., 2018), following which the abnormal mitochondria activities caused by I/R injury produce a large amount of ROS (Hsu et al., 2017; Wu et al., 2018) that causes cell apoptosis (Kim et al., 2018) or necrosis (Andrabi et al., 2017; Ji et al., 2017). Interestingly, folk remedies are derived from animal medicine, such as earthworm, hirudo, and *Zaocys dhumnades* conus. The secretions of these animals have also shown to have pharmacological activity; for instance, related peptides secreted by earthworm (Cornwell and Krajniak, 2014), hirudin secreted by hirudo (Ren et al., 2021), and conotoxin secreted by conus (AlSharari et al., 2020). Similarly, MQ is a metabolite of MILF, which is also animal secretion from *Entamoeba histolytica*. Several studies have reported the neuroprotective effects of MQ (Jiang et al., 2016; Zhu et al., 2016), which are small molecules and cheaper to produce compared to MLIF. The TTC staining results and infarct volume measurements did not detect any significant difference in the neuroprotection efficacy between MQ and MLIF. MQ exerts an antioxidant effect by decreasing the MDA and increasing the SOD serum content. Simultaneously, MQ significantly downregulated the apoptosis rate and ROS level compared to the OGD/R group.

Apoptosis is programmed cell death, and the process is an energy-dependent manner (D’Arcy, 2019; Farhood et al., 2019). In the early stage of acute ischemia, apoptosis may be a protective reaction of OGD, which maintains the survival of vital cells. However, calcium overload and the release of oxygen free radicals and lysosomal enzymes cause cell death during ischemia (Shi et al., 2015; Secondo et al., 2018; Yang, 2018). Furthermore, Caspase3 is an apoptosis-associated enzyme (Zheng et al., 2019) that plays a key role in cell apoptosis. Cerebral ischemia triggers the release of cytochrome c (cytC) from the mitochondria and promotes the activation of endogenous Caspase3 and apoptosis (Li et al., 2017; Jin et al., 2019; Wang et al., 2019). The two different forms of Caspase3, inactive and active (cleaved Caspase3), were used as the indexes of apoptosis because the expression of cleaved Caspase3 was elevated in the process of cell death (Adams and Cory, 1998). Conversely, the expression of cleaved Caspase3 was decreased in the An-Dong-Niu-Huang-Wan group compared to the MCAO group in ischemia (Wang et al., 2014). The current data showed that MQ treatment downregulated the expression of cleaved Caspase3.

The mitogen-activated protein kinase (MAPK) family proteins are serine-threonine kinases (Wang et al., 2017). The MAPK family mainly consists of c-Jun N-terminal kinases (JNKs), extracellular signal-regulated kinases (ERKs), and P38 (Wang et al., 2017; Chen et al., 2018). ERK1/2 upregulates B cell lymphoma 2 (Bcl-2), JNK regulates the transcription-dependent apoptotic signals to promote cell survival, and p38 is implicated in cell death, cell cycle, senescence, and carcinogenesis (Naoi et al., 2019). The MAPK pathway is triggered by various stimuli and physiological responses, including cell proliferation, differentiation, growth, inflammation, and apoptosis in mammalian cells (Zhang and Liu, 2002). The JNK signaling pathway plays a major role in the CNS, including the regulation of apoptosis and regeneration of both neuronal and glial cells (Salman et al., 2017). A recent study showed that the activation of JNK signaling pathways contributes to oxidative stress in ischemic kidney cell death (Yang et al., 2016). Additionally, the expression of JNKs, ERKs, and P38 and p-JNK, p-ERK, and p-P38 of the MAPK pathway is elevated in the cerebral ischemia mammalian brain model (Ferrer et al., 2003). Our preliminary experiment confirmed that MLIF inhibits the p-JNK/p53 pathway that exerts neural protection (Yao et al., 2011). Based on previous studies, we investigated the effect of MQ on p-JNK expression. The findings demonstrated that the level of p-JNK was markedly downregulated and further reduced by inhibiting the JNK activity with SP600125. The Bcl-2 protein family consists of anti-apoptotic (Bcl-2 and Mcl-1) and proapoptotic (Bax and Bad) molecules (Adams and Cory, 1998; Ruiz et al., 2018). Additional studies confirmed that Bcl-2 protein family adjusts the mitochondrial pathway by controlling the permeability of the outer membrane of the organelle (Sharma et al., 2019). The Bcl-2 proteins are localized in the mitochondria, endoplasmic reticulum, and nuclear membrane and are effective cell apoptosis inhibitors. Although Bax protein exists in the cytoplasm and endothelial surface (Singh et al., 2019), a configurational modification transfers it to the mitochondrial membrane as a response to apoptotic stimuli, such as the activation of Caspase (Bruel et al., 1997; Wolter et al., 1997; Gross et al., 1998). However, the apoptosis adjustment mechanism of Bcl-2 and Bax is not yet clarified. Jin et al. reported that simvastatin alleviated spleen atrophy and spleen cell apoptosis caused by stroke via increased protein expression of Bcl-2 and reduced level of Bax (Jin et al., 2013). In this study, we found that Bax, a JNK downstream protein, plays a role in the mechanism of cerebral ischemia after MQ treatment.

Overall, the current study showed that MQ reduced neurological dysfunction, neuronal injury, cerebral infarction, and the MDA level and increased the SOD level in a rat model, which might be associated with the downregulated oxidation processes. This finding indicates that the treatment of MQ exerts antioxidant and anti-apoptotic effects, rendering it beneficial for stroke because it prevents reperfusion-induced injury. Nevertheless, the current study has some limitations. We studied the protective effect of I/R injury; thrombolysis was not involved as the main treatment. Importantly, MQ can be proposed as an auxiliary treatment as a part of rehabilitation after thrombolysis. Future studies are expected to design therapeutic medicine based on MQ for controlling cerebral I/R injury.

## Data Availability

The raw data supporting the conclusions of this article will be made available by the authors, without undue reservation, to any qualified researcher.
